# In Vitro Evaluation of the Antibacterial Properties and Cellular Response of Liquid-Leukocyte Platelet-Rich Fibrin Products on Barrier Membranes: A Pilot Study

**DOI:** 10.3390/dj13060228

**Published:** 2025-05-22

**Authors:** Nichol Chun Wai Tsang, Aneesha Acharya, Georgios Pelekos

**Affiliations:** 1 Division of Periodontology and Implant Dentistry, Faculty of Dentistry, The University of Hong Kong, Hong Kong SAR, China; nichol@connect.hku.hk (N.C.W.T.); aneesha.acharya@dpu.edu.in (A.A.); 2 Dr. D. Y. Patil Dental College & Hospital, Dr. D. Y. Patil Vidyapeeth, Pimpri, Pune 411018, India

**Keywords:** liquid platelet-rich fibrin, antimicrobial membrane treatment, guided bone regeneration, liquid fibrinogen, resorbable membranes, antibacterial properties, dental surgery

## Abstract

**Background:** Barrier membranes (BMs) have been used in dental surgical procedures for decades, but their exposure can increase the risk of infections and compromise healing from regenerative procedures. Liquid-leukocyte platelet-rich fibrin (LPRF) products have shown antimicrobial effects and enhance wound healing. This in vitro study aimed to evaluate the antimicrobial effects and cellular responses of LPRF products as adjunctive treatments for barrier membranes, hypothesizing that the two liquid LPRF products could improve antibacterial activity against selected oral pathogen species and augment human gingival fibroblast cellular proliferation on BM. **Methods:** LPRF exudate (LPRF-EX) and liquid fibrinogen (PLyf), human LPRF products, were prepared with recommended centrifugation protocols and used to treat resorbable (Bio-gide^®^) and non-resorbable (Cyto-plast™) BMs. Human gingival fibroblasts (HGFs) were cultured on the treated and untreated BMs. Scanning electron microscopy (SEM) was applied to observe cell adhesion, and CCK-8 assays were used to study cell proliferation. Oral *P. gingivalis* and *A. naeslundii* were incubated with the BMs. Bacterial adhesion was visualized using SEM, and colony-forming unit (CFU) counts were obtained. **Results:** SEM images showed markedly greater fibrin network formation after 7 days on resorbable BM (Bio-gide^®^) treated with PLyF, but with no notable differences in other resorbable BM or non-resorbable BM groups with both treatments. CCK-8 assays showed non-significant effects on HGF proliferation at 3 and 5 days. SEM showed *A. naeslundii* growth inhibition in the LPRF-EX- and PLyf-treated BMs, and the greatest reduction in CFU counts of both *P. gingivalis* and *A. naeslundii* was noted with treated Cytoplast™. **Conclusions:** Within the limitations of this preliminary study, it can be concluded that the LPRF-EX and PLyf treatment of BM induced an antimicrobial effect. Their effects on cellular response were unclear due to the lack of significant findings on SEM analysis.

## 1. Introduction

Several biomaterials, scaffolds, biomodulators, and other approaches have been developed and introduced for use in regenerative dentistry, including the use of genes, stem cells, bone substitutes, and growth factors [1]. Regenerative dentistry has stimulated the development of several biomaterials for use in various techniques such as sinus augmentation, ridge preservation, guided bone regeneration (GBR) and periodontally regenerative procedures [2]. Amongst these, platelet-rich plasma (PRP) was first introduced in the mid-1990s and is commonly regarded as a first-generation platelet concentrate. PRP, derived from whole blood, promotes healing in the maxillofacial area owing to its presence of growth factors [3,4]. However, the preparation of PRP is a costly and relatively complicated procedure. Leukocyte- and platelet-rich fibrin (LPRF), also known as a second-generation platelet concentrate, was developed in 2001, and is in gel form, with a much more straightforward and cost-effective preparation procedure [5]. It involves a single centrifugation step [6], and this simplified procedure also minimizes the risk of trans-contamination. Blood is collected in vacuum tubes and subsequently centrifuged at a standard rate, producing three layers, including a fibrin-rich LPRF clot [5]. Further evolution of PRF products has included advanced PRF (A-PRF) and advanced PRF plus (A-PRF+), which involve longer centrifugation times [7].

The LPRF clot is formed after centrifugation, and three layers are separated: the platelet-poor plasma as the first layer (PPP); a fibrin clot (i.e., LPRF) forming the second layer, which is used in surgical procedures; and red blood cells forming the third layer [5]. The fibrin clot is a three-dimensional fibrin network with a thin whitish layer in between termed the “buffy coat”, which corresponds to the accumulated platelets trapped in the PRF matrix [8,9]. Following the centrifugation, different types of cells are trapped within this three-dimensional matrix, including platelets, leukocytes, macrophages, granulocytes, and neutrophils. The incorporation of white blood cells, especially leukocytes, also adds antibacterial and immune regulatory properties to LPRF [10]. The LPRF clot contains several growth factors, such as platelet-derived growth factor (PDGF), vascular endothelial growth factor (VEGF), and transforming growth factor (TGF), which contribute to accelerated soft and hard tissue healing [11] by facilitating rapid proliferation, regulating cell migration, and promoting angiogenesis. In addition, osteogenic cells are also found in the LPRF, which can differentiate to osteocytes and osteoblasts and facilitate hard tissue healing [12]. Antimicrobial effects of LPRF preparations have been attributed to the release of antimicrobial peptides, peroxide, and biofilm inhibition [13], and liquid LPRF has shown improved antimicrobial effects.

A lower centrifugation force can cause more leukocytes to be trapped in the PRF matrix [14]. Thus, modifications of centrifugation speed and time have a significant impact on the type and number of cells and growth factors, as well as the fibrin architecture of the LPRF clot [15]. Several LPRF products have been reported using such modifications. During the transformation of an LPRF clot to membrane by compression, LPRF exudate (LPRF-EX) is produced as a by-product. The exudate derived is rich in various plasma proteins, such as fibronectin [16], and angiogenic growth factors and proteins [16]. By shortening the centrifugation time, another platelet concentrate can be produced, namely liquid fibrinogen (PLyF) [15]. This liquid form of concentrate contains platelets, leukocytes, plasma proteins, and fibrinogen, with an almost 1.5-fold mean accumulation of platelets along with slow release of various growth factors such as TGF-β1, PDGF-AB, FGF-2, and VEGF [15,17]. Other LPRF products include injectable platelet-rich fibrin [18,19] and advanced platelet-rich fibrin (A-PRF), produced by adjusting the centrifugation time and rate.

Guided bone regeneration (GBR) involves the creation of a mechanical barrier that prevents the soft tissue from growing into an osseous defect, thus allowing osteoprogenitor cells to proliferate and differentiate such that osteogenic cells can repopulate the wound [20]. A number of barrier membranes (BMs) have been developed, serve to “guide” the type of tissue in the healing site by functioning as a barrier against epithelial tissue, and can include resorbable and non-resorbable BMs. Resorbable BMs usually comprise collagen, which may be of xenogeneic or human origin [21].

The most frequently encountered clinical complication in the use of BMs remains their exposure, which may lead to infection and compromised wound healing. While the rate of non-resorbable BM exposure varies across clinical studies, it is well documented, and complication rates of up to 45.5% are reported in cases of their application in vertical bone augmentation [22,23]. When BMs are exposed, oral bacteria rapidly colonize the membrane, which can subsequently cause infection and necessitate early membrane removal [24]. The colonizing species and biofilm formation vary depending on the type and composition of the BM [25]. BM exposure raises the risk of infection and compromised healing, potentially leading to a negative effect on the outcome of GBR (guided bone regeneration) and GTR (guided tissue regeneration) [25,26,27]. BM exposure is associated with substantially lower bone gain, with 74% lower gain reported in edentulous ridge augmentation [28]. A meta-analysis reported a 22.7% complication with resorbable BMs in vertical ridge augmentations [29]. Commonly used BMs do not possess antibacterial properties [26]. BMs can act as carriers of bioactive molecules [30,31], and as the antibacterial property of BMs can be very advantageous, attempts to induce antibacterial effects in BMs have included incorporation of antibiotics, antimicrobial peptides, and metallic nanoparticles, but antibiotics in particular raise concerns of antimicrobial resistance and potential cytotoxicity [32]. Clinical evidence supporting the effectiveness of antimicrobial BM treatments is very scarce [33]. Taken together, these findings point to a need for bifunctional BM treatments that confer both antimicrobial and regenerative properties. Considering these properties of LPRF products, they could be attractive adjuncts to BMs.

The specific aims of this study were to characterize the antimicrobial effects and cellular responses induced by two LPRF products: LPRF-EX and PLyF treatment of resorbable and non-resorbable barrier membranes. We hypothesized that the two liquid LPRF products could improve antibacterial activity and enhance human gingival fibroblast cellular proliferation on barrier membranes. To the best of our understanding, the effects of different LPRF products as an additive treatment for BMs are yet to be investigated. Therefore, we hypothesized that liquid LPRF products can be used as a treatment of resorbable and non-resorbable barrier membranes, Bio-gide^®^ (BG) and Cytoplast™ (CP), to provide a simple means of inducing an antimicrobial effect while improving wound healing-related cellular events. The results could clinically translate to the reduced incidence of bacterial infection and compromised healing and improved wound healing properties in cases of BM exposure.

## 2. Results

### 2.1. HGF Cell-Culture Experiments

#### 2.1.1. SEM Analysis of Untreated BM

**Figure 1:**
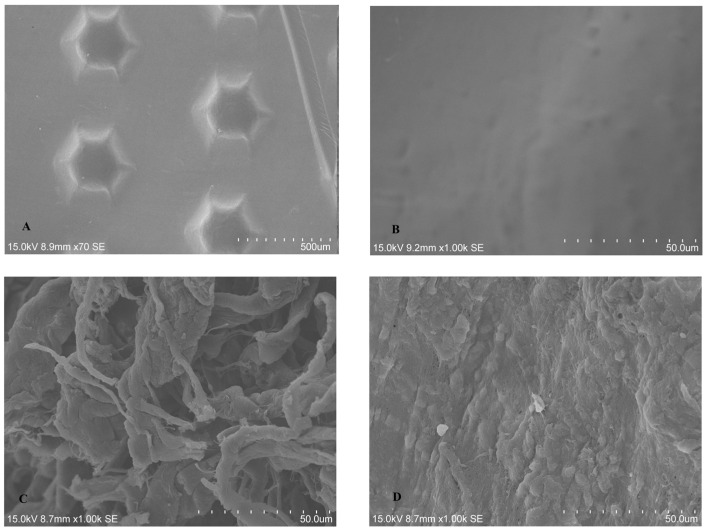
SEM images showing (**A**) hexagonal surface of CP; (**B**) smooth surface side of CP; (**C**) the rough, high-porosity surface of BG; and (**D**) the smooth cell occlusive surface of BG.

The two BMs displayed distinct appearances under SEM. The CP showed hexagonal surface, which purportedly increases the area available for cellular attachment without increasing porosity, whereas coarse fibers were evident on the rough surface of BG, which has high similarities to human collagen (Figure 1A–D).

#### 2.1.2. SEM Analysis of Treated Membrane with HGF Cell Culture

**Figure 2:**
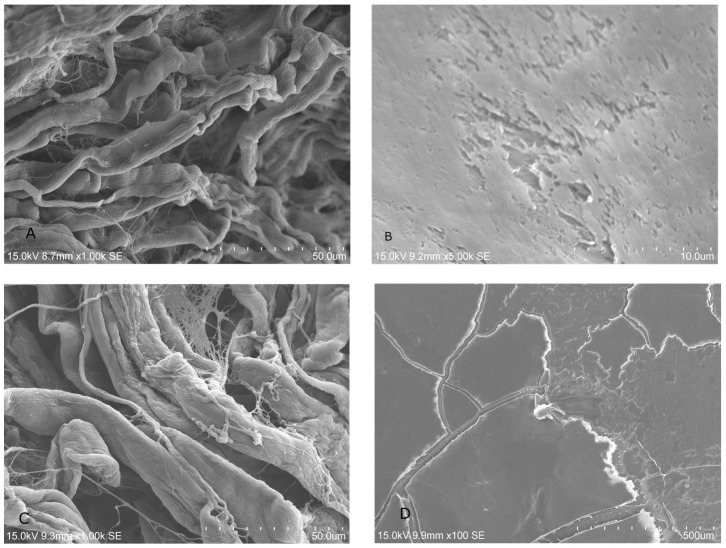
SEM images of HGF cultures on day 3: (**A**) treatment of LPRF-EX on BG; (**B**) treatment of LPRF-EX on CP; (**C**) treatment of PLyF EX on BG; (**D**) treatment of PLyF on CP.

**Figure 3:**
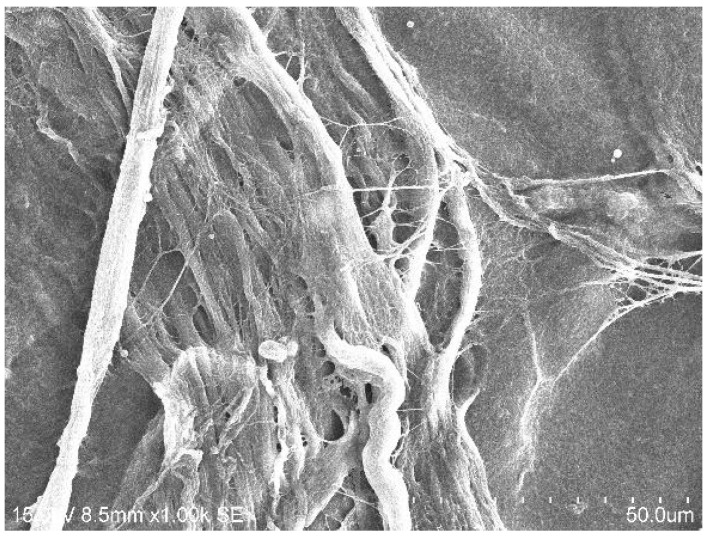
SEM image showing HGF proliferation on BG treated with PLyF on day 7.

The treatment with LPRF-EX (Figure 2A,B) showed no obvious changes in cell growth on either BM after treatment. SEM showed robust fibrin network on BG treated with PLyF on day 7 (Figure 3).

#### 2.1.3. HGF Cell Proliferation Assays

**Figure 4:**
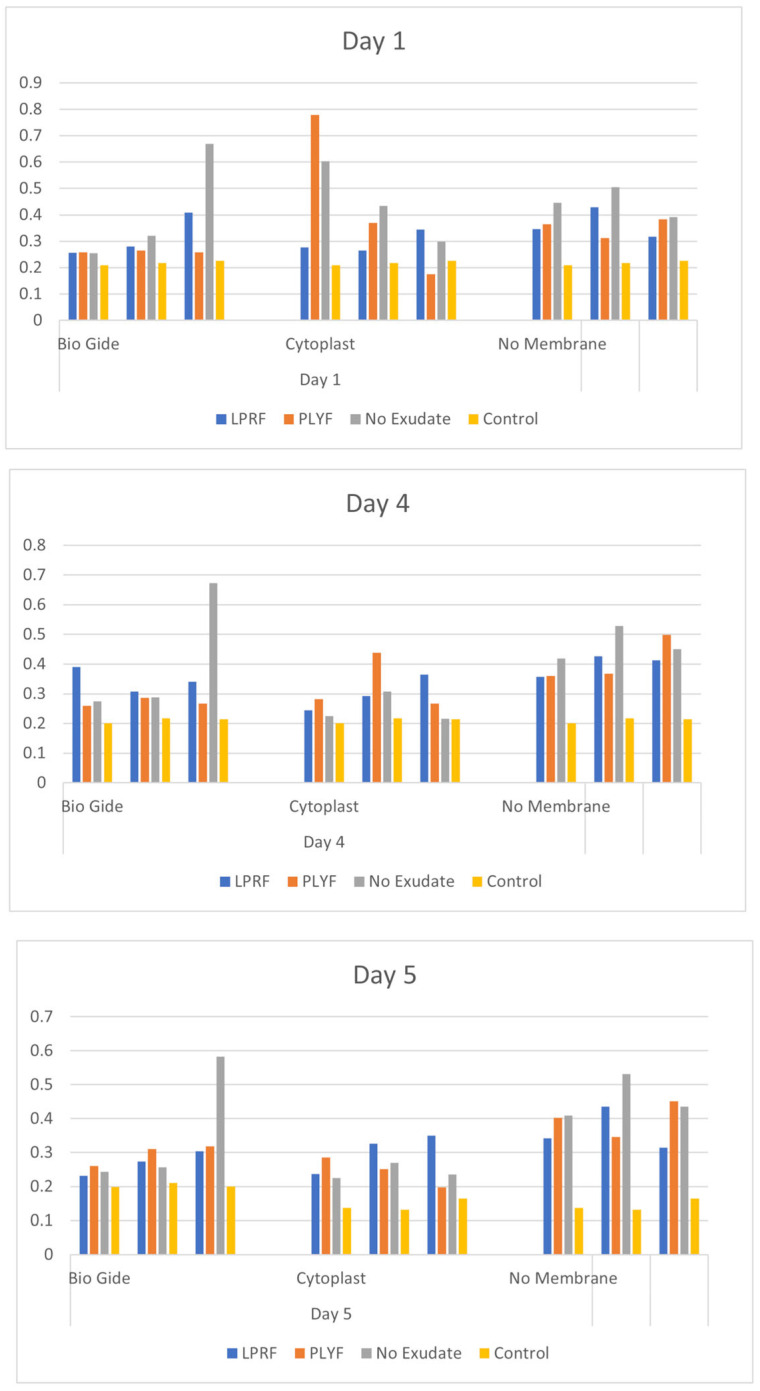
Bar plots depicting HGF cell proliferation represented by CCK-8 assay absorbance values on days 1, 3, and 5.

Results of HGF cell proliferation investigated with CCK-8 (Cell Counting Kit-8) assays are depicted in Figure 4. No significant intergroup differences were found. Trend analysis showed that in the absence of membrane and/or LPRF products, cell proliferation appeared as the highest. A trend for greater cell proliferation at CP treated with PLyF on day 1 (Figure 4) was evident, suggesting a rapid release of growth factor at the initial stage.

Conversely, a trend indicating enhanced proliferation of HGF cells under LPRF-EX was noted at day 3 and day 5 (Figure 4), suggesting a potential slow release of growth factor. However, upon statistical analysis, no significant difference was noted in cell proliferation between different LPRF treatments (LPRF-EX or PLyF) (Table 1). Further studies extending the duration of investigation may yield more conclusive results.

### 2.2. Oral Bacterial Culture Experiments

#### 2.2.1. Bacterial Adhesion on BMs Examined by SEM

**Figure 5:**
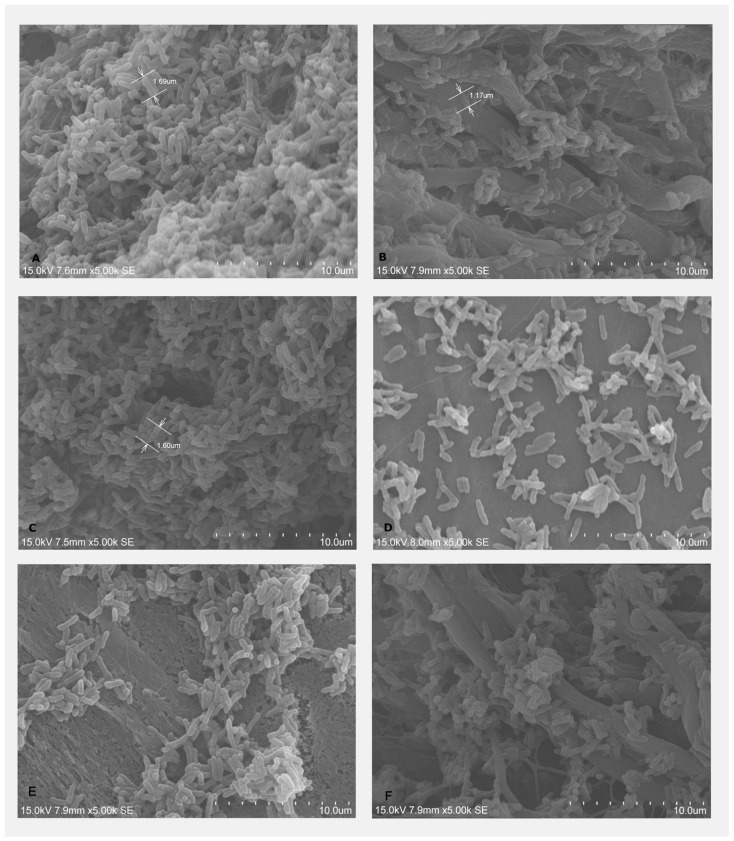
SEM images of A. naeslundi culture on: (**A**) untreated BG; (**B**) untreated CP; (**C**) LPRF-EX-treated BG; (**D**) LPRF-EX-treated CP; (**E**) PLyF-treated BG; (**F**) PLyF-treated CP.

Bacterial adhesion of *A. naeslundii* on the treated and untreated BMs was visualized under SEM, showing that *A. naeslundii* could adhere to both BG and CP. BM treated with LPRF-EX demonstrated fewer adherent cells. The subjective assessment of quantity of adherent bacterial cells appeared lower on the BG treated with PLyF (Figure 5E) than that of BG treated with LPRF-EX (Figure 5C). Overall, it was apparent that the adherent bacteria decreased, demonstrating the possible antimicrobial effects of PLyF and LPRF-EX on *A. naeslundii* growth on BMs.

#### 2.2.2. Bacterial CFU Counts

**Figure 6:**
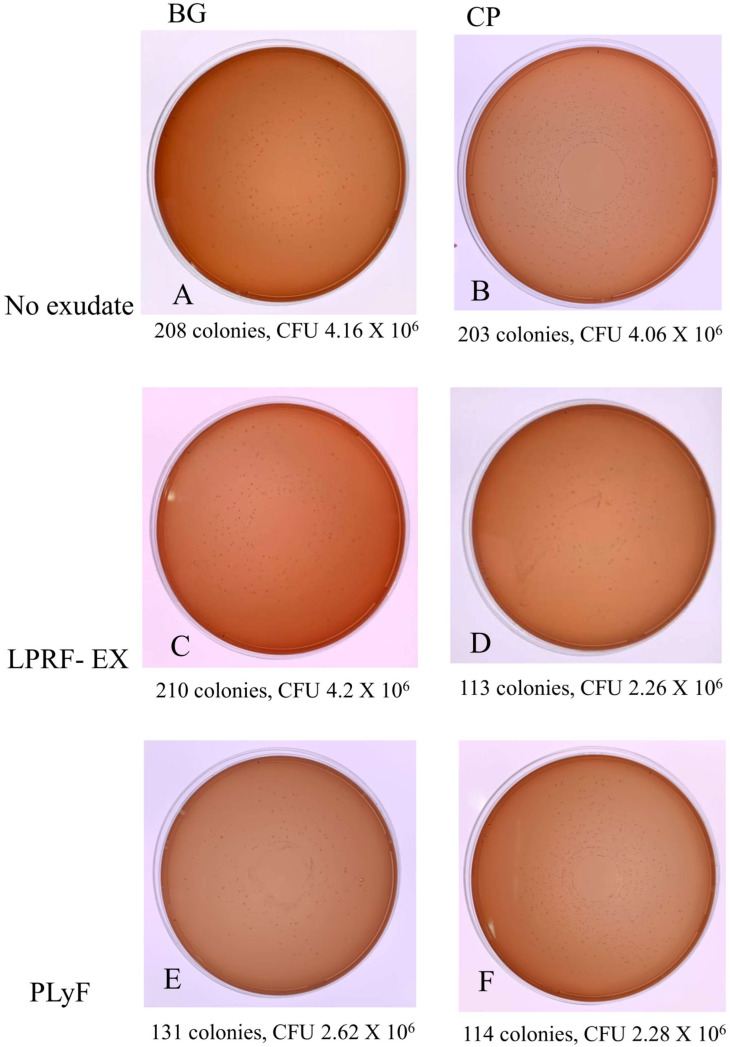
CFU counts of *A. naeslundi* culture on: (**A**) untreated BG; (**B**) untreated CP; (**C**) LPRF-EX-treated BG; (**D**) LPRF-EX-treated CP; (**E**) PLyF-treated BG; (**F**) PLyF-treated CP.

**Figure 7:**
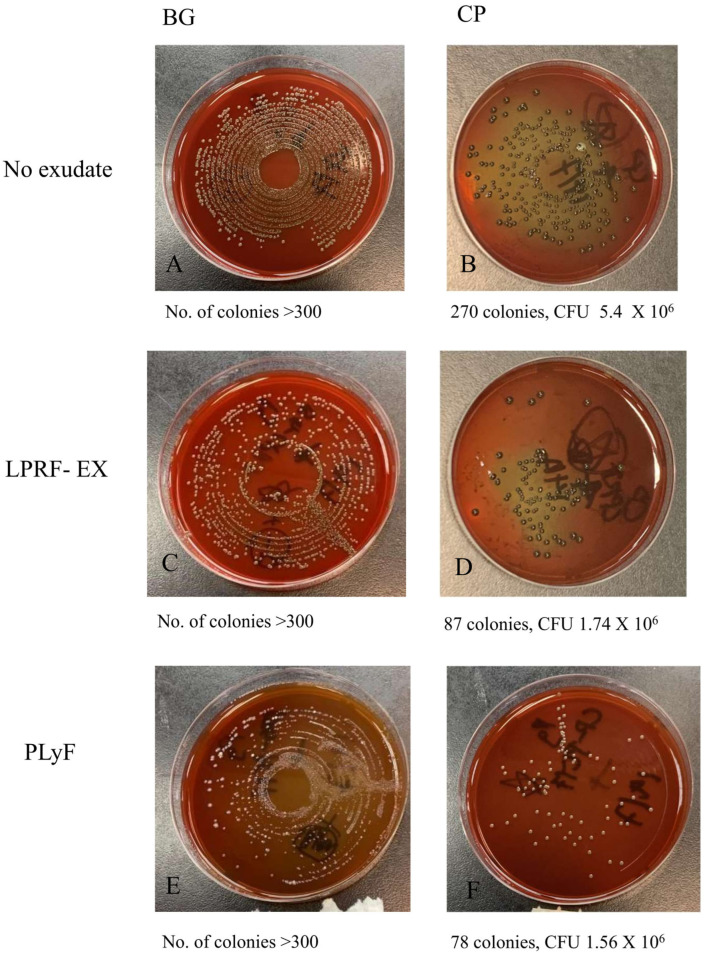
CFU counts of *P. gingivalis* culture on: (**A**) untreated BG; (**B**) untreated CP; (**C**) LPRF-EX-treated BG; (**D**) LPRF-EX-treated CP; (**E**) PLyF-treated BG; (**F**) PLyF-treated CP.

From the results of CFU counts (Figure 6 and Figure 7), it was apparent that the treatment with LPRF-EX or PLyF on both BMs produced antibacterial properties, in which the inhibitory effect of LPRF-EX on *A. naeslundii* growth on CP was the most marked.

## 3. Discussion

Within its limitations, the present study provides preliminary in vitro evidence suggestive of antimicrobial effects of the two different LPRF products when used in combination with resorbable and non-resorbable barrier membranes, while biomodulator effects were unclear. A dose-dependent effect of LPRF on HGF proliferation has been shown [34,35]. The comparison of two different LPRF products and two different barrier membranes has not been reported until now. LPRF enhances bone augmentation outcomes [36]. Clinically, liquid LPRF applied in combination with bovine-derived xenograft improved GBR outcomes in peri-implant bone augmentation [37]. Preclinical animal histological data have shown that the coating of Biogide^®^ with LPRF improved new bone formation [38]. The consistency of the findings across the treatment groups verified the primary research assumptions while raising the need for studies to optimize specific LPRF treatment protocols for improved outcomes. Others have shown differences in penetration depth of different collagen membranes biofunctionalized by liquid LPRF products [39] and improved angiogenesis [40], further highlighting the need for investigations focused on specific BM and LPRF preparation combinations.

Antimicrobial activity was shown in this study: the growth and adhesion of *A. naeslundii* was inhibited by the LPRF treatments. A more marked inhibitory effect upon *P. gingivalis* and *A. naeslundii* growth was seen on CP, suggesting a potentially greater benefit. The two oral species chosen, *A naeslundi* and *P. gingivalis*, are implicated in periodontitis as an early colonizer [41] and keystone species [42], respectively. The antimicrobial effect of LPRF products has been attributed to the increased release of oxygen metabolites and leucocytes in previous work that showed iPRF (injectable platelet-rich fibrin, other forms of LPRF products) had greater antimicrobial activity against *P. gingivalis* [43,44].

In addition to the antimicrobial properties, we assessed the early-phase cell response during the first week and quantified the HGF proliferation on days 1, 3, and 5, representing initial seeding and proliferation [45,46]. SEM analysis showed an increased presence of fibrin networks on BG treated with PLyF at 3 and 7 days. The resorbable BM treated with PLyF showed denser fibrin network formation by cultured HGFs compared to the control, suggestive of a potential increase in cellular activity.

However, the differences in HGF proliferation were insignificant, pointing to the need for longer-term and larger-sample studies. A trend for greater cell proliferation for CP treated with PLyF on day 1 was evident and may suggest a rapid release of growth factors in initial stages that may have led to greater early HGF proliferation, whereas the trend for increased proliferation of HGF cells under LPRF-EX on days 3 and 5 may indicate a slower growth factor release. The characterization of growth factor release from PLyF showed increased levels of TGF-β1, PDGF-AB, FGF-2, and VEGF levels over 7 days, with sustained high release of PDGF-AB till 14 days, which are key growth factors in wound healing [15]. LPRF-EX can stimulate platelet activation and promote wound healing and immune modulation via an interleukin 8-dominated cytokine response [47]. Liqiud LPRF treatment shows different chemokine gene expression patterns for HGF compared to PRF clots [48]. Comparative molecular investigations are warranted to understand the biological mechanisms underlying host cell responses to BMs treated with various LPRF preparations.

Taken together, these preliminary findings support the positive biofunctional and antimicrobial effects of LPRF preparations as treatments for both resorbable and non-resorbable barrier membranes (BMs), broadly aligning with previous findings. Liquid LPRF treatment of implant surfaces has been shown to promote fibrin network formation [49]. Blatt et al. [50] showed increased pro-angiogenesis and vessel growth on CP treated with liquid PRF. Specifically, we showed that the application of LPRF-EX and PLyF can confer antimicrobial effects and enhance cellular wound healing processes, which may clinically translate to a reduced incidence of bacterial infections and compromised healing in cases of barrier membrane exposure. The antibacterial potential of liquid LPRF products may have important clinical relevance in regenerative dentistry. The improved wound healing properties could be particularly valuable for patients with systemic conditions, such as diabetes, or in areas with a high risk of infection. Clinical research that investigates the benefits of the putative antimicrobial property of these treatments in specific subpopulations such as diabetics and immunocompromised and geriatric patients and higher-risk procedures such as vertical bone augmentation can comprise a future direction of research. Clinical implications of such findings could include LPRF treatment-based protocols of GBR and GTR that reduce the incidence of BM complications and enhance clinical outcomes.

Major limitations of the study include the small sample and duration of the study, which warrant validation in larger-sample and longitudinal studies. The lack of in vivo validation and potentially divergent clinical effects also represent key limitations. No sample size estimation was performed, and the results may be considered preliminary and warrant verification. The present study did not include any molecular analysis and SEM provided qualitative data, which can be considered preliminary. Given the nature of in vitro study, there could be variability in clinical outcomes. Further research is warranted to establish the differences in biological effects produced by various LPRF products besides LPRF-EX and PLyF and potential clinical trials to enable the selection of optimal clinical protocols for the biofunctionalization of barrier membranes and assess the short- and long-term effects clinically. Moreover, specific LPRF preparation methods should be investigated further, and the effects of centrifuge features, protocols, donor traits, and the downstream effects on wound healing warrant greater elucidation. Clinical trials that assess the long-term effects of these LPRF-based treatments on wound healing and treatment outcomes are essential.

## 4. Materials and Methods

### 4.1. Subject Recruitment, Inclusion and Exclusion Criteria

Blood was collected from the antecubital vein of 4 healthy volunteers recruited as a convenience sample, with standard aseptic precautions after obtaining informed consent. The inclusion criterion was healthy males or females aged 20–45 years. The exclusion criteria were (i) pregnancy or lactation; (ii) diagnosed medical conditions or diseases (e.g., diabetes); (iii) hematological diseases or bleeding disorders (e.g., platelet dysfunction or thrombocytopenia); (iv) being on anticoagulants or antiplatelet drugs; and (v) a history of smoking or alcohol consumption. These conditions were excluded, as they may affect changes in the clotting mechanisms and platelet regulatory pathways. The study protocol was reviewed and approved by the Institutional Review Board of the University of Hong Kong. Written informed consent was obtained from the included subjects undergoing blood collection procedures.

### 4.2. Preparation of L-PRF Products

All L-PRF products were prepared using the same centrifuge system (IntraSpin™, Intra-Lock, Boca Raton, FL, USA). For, LPRF-EX, 4 tubes of 9 mL of venous blood were collected by the same investigator (N.C.W.T) into red-capped vacuum tubes, transferred to the centrifuge machine, and centrifuged under a standardized protocol at 12-min and 2700 rpm to firstly obtain LPRF clots (Figure 2A). The LPRF clots were collected with surgical tweezers and subsequently transferred to a sterile box (Xpression™ Kit, Intra-Lock, Boca Raton, FL, USA) (Figure 2B). The clots were compressed in the kit (Figure 2C) for 5 min to form LPRF membranes, and the resulting exudate was LPRF-EX, which was collected with a sterile syringe.

Similarly to the preparation of LPRF-EX, 4 tubes of 9 mL venous blood were collected into white-capped vacuum tubes and centrifuged with a different protocol, 3 min centrifugation at 2700 rpm, as reported earlier [34]. Under this centrifugation rate, the yellow fluid (PLyF) at the top layer was carefully aspirated using a sterile syringe (5 mL) and 18-gauge needle (Figure 3).

The LPRF-EX and PLyF were brought to the laboratory and filtered with a syringe filter collected and prepared for use to treat the BMs. All procedures were performed under a tissue culture hood.

Centrifuge specific features and centrifugation protocols are known to affect the cell concentration and growth factor amounts and timing of release [35], which may have precluded the exact replication of previously reported protocols.

### 4.3. Preparation and Treatment of BMs

Bio-gide^®^ (BG) (Figure 5A) (Geistlich Pharma AG, Wolhusen, Switzerland) and Cytoplast™ (CP) (Osteogenics Biomedical, Lubbock, TX, USA) (Figure 5B) BMs were sectioned into 4.7 mm circular sections using a sterile mucosa punch (Straumann Mucosa punch Ø 4.7 mm, guided—L 30 mm, Figure 5C) with finger pressure. These Bio-gide^®^ and Cytoplast™ BM specimens were placed in 24-well plates or 96-well plates. The smooth, cell-occlusive, low-porosity compact surface was oriented facing inwards, and the rough, high-porosity, spongy surface was oriented facing outwards. Each membrane was handled aseptically upon the sealing package using sterile tweezers, prepared using a mucosal punch (Straumann™, Basel, Switzerland), and placed directly into culture plates under the laminar hood.

The sectioned BM specimens were randomized and treated by 10 min inoculation with test treatments: (i) LPRF-EX; (ii) PLyF; and control (iii) Dulbecco’s modified Eagle’s medium (DMEM) (Thermofisher Scientific^®^, Waltham, MA, USA). DMEM is a standard cell-culture medium and most commonly used for gingival fibroblast culture and was hence chosen as the control reagent [36]. The treatment media were added till the BM was completely submerged.

### 4.4. Human Gingival Fibroblast (HGF) Cell-Culture Experiments

#### 4.4.1. Cells and Culture

A human gingival fibroblast 1 cell line (PCS-201-018, ATCC^®^ CRL-2014™, Manassas, VA, USA) (HGF-1) was procured from ATCC^®^ and cultures from passage 4–6 were used. Cells were seeded on treated BM specimens at a seeding density of 2 × 103 cells/cm^2^ in 24-well plates for scanning electron microscopy (SEM) or 96-well plates (for CCK8-assays), and immersed in DMEM supplemented with 5% fetal bovine serum and 1 mL of antibacterial agent (Primocin^®^, InvivoGen, San Diego, CA, USA) (Figure 6A,B). Culture plates were placed within a humidified atmosphere (95% air, 5% carbon dioxide) and incubated at 37 °C. After 4 h, the seeded specimens were washed with phosphate-buffered saline to remove non-adherent cells and transferred to new wells. Culture media were changed at 24 h, then every 48 h. Cell seeding density was selected in accordance with ATCC recommendations.

#### 4.4.2. Scanning Electron Microscopy for Fibroblast Adhesion

SEM was performed on day 3 and day 7. Specimens collected on day 3 included (i) BG with no treatment; (ii) CP with no treatment; (iii) BG with LPRF-EX ± HGF; (iv) BG with PLyF ± HGF; (v) CP with LPRF-EX ± HGF; and (vi) CP with PLyF ± HGF. Specimens were fixed with 2.5% glutaraldehyde overnight, followed by dehydration on the following day in graded alcohol series (70%, 85%, 95% and 100%). The specimens were stored in a desiccator at room temperature and processed to visualize under SEM. The same procedures were repeated on day 7.

#### 4.4.3. Cell Proliferation Assays

Cell Counting Kit-8 (CCK-8, Dojindo Laboratories^®^, Kumamoto, Japan) colorimetric assays were used to study cell proliferation. Cell proliferation was studied on day 1, day 3 and day 5. Old culture medium was discarded, and new fresh medium (DMEM with 5% FBS and antibacterial agent) containing diluted CCK-8 reagent (10 µL CCK8 and 100 µL fresh medium) was used to incubate the specimens for 3 h. The solution was transferred to a new 96-well plate with micropipettes for measuring absorbance at 450 nm. A new culture medium was added to the original 96-well plates with BMs. All experiments were performed in triplicate: BG treated with LPRF-EX; BG treated with PLyF; BG with no treatment; CP treated with LPRF, CP treated with PLyF; CP with no treatment; and a control with no BM (Figure 6A–C). All experiments were performed in triplicate (Figure 6).

### 4.5. Oral Bacterial Infection Experiments

#### 4.5.1. Bacterial Culture

Two selected oral microbial species, *P. gingivalis* (ATCC W83) and *A. naeslundii* (ATCC 12104), were procured from ATCC^®^ (Manassas, VA, USA), incubated with the treated specimens in 24-well plates for 2 days for *A. naeslundii* using BHI broth and 4 days for *P. gingivalis* using *P. gingivalis* broth, and cultured in an anaerobic chamber. Before the assays, the optical density of each culture was adjusted to 0.1.

#### 4.5.2. Scanning Electron Microscopy (SEM) (Hitachi™ VP-SEM SU1510, Hitachi, Tokyo, Japan) for Bacterial Adhesion

The treated specimens were collected at day 2 for *A. naeslundii* and day 3 for *P. gingivalis*. The specimens were rinsed with 0.9% sterile saline baths and fixed with 2.5% glutaraldehyde overnight (12 h), followed by dehydration the next day in increasing grades of alcohol (70%, 85%, 95%, and 100%) for 15 min each. Specimens were then stored in a desiccator at room temperature for 1 h and SEM was used to visualize adherent bacteria. Four representative areas were selected for assessment randomly and images were photographed at magnifications from 70 to 5000×.

#### 4.5.3. Colony-Forming Unit (CFU) Counts

The treated specimens collected were washed and sonicated for 5 min to detach the adherent microbes into a recovery medium. Serial dilutions were plated onto tryptic soy agar and incubated for another 2 to 4 days, and the numbers of colonies were counted.

### 4.6. Data Analysis

Qualitative analysis of the SEM images was performed by the same examiner. CFU counts were compared. The CCK-8 assay absorbance values were used to calculate the mean of triplicate experimental samples and two-way ANOVA with Bonferroni’s multiple comparison test was applied using Prism 9.4.0 software (GraphPad Software, San Diego, CA, USA). A *p*-value < 0.05 was considered statistically significant. Normality distribution was verified with the Shapiro–Wilk test.

## Figures and Tables

**Table 1:** Comparison of CCK-8 absorbance values for HGF proliferation on differently treated BMs. ns means no significant findings.

	Day 1			Day 3			Day 5		
Bonferroni’s Multiple Comparison Test	Mean Diff.		Adjusted *p* Value	Mean Diff.		Adjusted *p* Value	Mean Diff.		Adjusted *p* Value
BG									
LPRF vs. PLYF	0.0549	ns	0.8859	0.07477	ns	>0.9999	−0.02683	ns	>0.9999
LPRF vs. No Exudate	−0.09857	ns	0.6808	−0.0658	ns	>0.9999	−0.09123	ns	0.5255
PLYF vs. No Exudate	−0.1535	ns	0.4051	−0.1406	ns	0.2464	−0.0644	ns	0.9969
CP									
LPRF vs. PLYF	−0.1459	ns	0.4402	−0.0286	ns	>0.9999	0.05997	ns	>0.9999
LPRF vs. No Exudate	−0.15	ns	0.4211	0.05177	ns	>0.9999	0.06123	ns	>0.9999
PLYF vs. No Exudate	−0.0041	ns	0.9993	0.08033	ns	0.9202	0.001267	ns	>0.9999
No Membrane									
LPRF vs. PLYF	0.009933	ns	0.996	−0.0098	ns	>0.9999	−0.03547	ns	>0.9999
LPRF vs. No Exudate	−0.0844	ns	0.753	−0.0674	ns	>0.9999	−0.09417	ns	0.4871
PLYF vs. No Exudate	−0.09433	ns	0.7026	−0.0575	ns	>0.9999	−0.0587	ns	>0.9999

## Data Availability

The raw data generated in this study can be provided by the corresponding author upon reasonable request.

## Abbreviations

Bio-gide^®^ (BG); Cytoplast™ (CP); barrier membrane (BM); human gingival fibroblast (HGF); LPRF exudate (LPRF-EX); liquid fibrinogen (PLyf); platelet-rich fibrin (PRF); platelet-rich plasma (PRP); scanning electron microscopy (SEM).
